# Biosynthesis of the nosiheptide indole side ring centers on a cryptic carrier protein NosJ

**DOI:** 10.1038/s41467-017-00439-1

**Published:** 2017-09-05

**Authors:** Wei Ding, Wenjuan Ji, Yujie Wu, Runze Wu, Wan-Qiu Liu, Tianlu Mo, Junfeng Zhao, Xiaoyan Ma, Wei Zhang, Ping Xu, Zixin Deng, Boping Tang, Yi Yu, Qi Zhang

**Affiliations:** 1 Jiangsu Key Laboratory for Bioresources of Saline Soils, Jiangsu Synthetic Innovation Center for Coastal Bioagriculture, Yancheng Teachers University, Yancheng, 224002 China; 2 Department of Chemistry, Fudan University, Shanghai, 200433 China; 3 Ministry of Education Key Laboratory of Cell Activities and Stress Adaptations, School of Life Sciences, Lanzhou University, Lanzhou, 730000 China; 4 Key Laboratory of Extreme Environmental Microbial Resources and Engineering, Northwest Institute of Eco-environment and Resource, Chinese Academy of Sciences, Lanzhou, 730000 China; 5 State Key Laboratory of Proteomics, National Center for Protein Sciences, Beijing Proteome Research Center, Beijing Institute of Radiation Medicine, Beijing, 102206 China; 6 Key Laboratory of Combinatory Biosynthesis and Drug Discovery (Ministry of Education), School of Pharmaceutical Sciences, Wuhan University, Wuhan, 430071 China

## Abstract

Nosiheptide is a prototypal thiopeptide antibiotic, containing an indole side ring in addition to its thiopeptide-characteristic macrocylic scaffold. This indole ring is derived from 3-methyl-2-indolic acid (MIA), a product of the radical *S*-adenosylmethionine enzyme NosL, but how MIA is incorporated into nosiheptide biosynthesis remains to be investigated. Here we report functional dissection of a series of enzymes involved in nosiheptide biosynthesis. We show NosI activates MIA and transfers it to the phosphopantetheinyl arm of a carrier protein NosJ. NosN then acts on the NosJ-bound MIA and installs a methyl group on the indole C4, and the resulting dimethylindolyl moiety is released from NosJ by a hydrolase-like enzyme NosK. Surface plasmon resonance analysis show that the molecular complex of NosJ with NosN is much more stable than those with other enzymes, revealing an elegant biosynthetic strategy in which the reaction flux is controlled by protein–protein interactions with different binding affinities.

## Abstract (web summary)

Thiopeptides such as nosiheptide are clinically-interesting antimicrobial natural products. Here the authors show the functional dissection of a series of enzymes involved in nosiheptide biosynthesis, revealing a unique biosynthetic pathway that centers on a previously-unknown carrier protein.

## Introduction

Ribosomally synthesized and posttranslationally modified peptides (RiPPs) are a major class of natural products, as revealed by the genome-sequencing programs of the past decade^1, 2^. These compounds are found in all three domains of life, possessing vast structural diversity ranging from relatively simple and linear structures as exemplified by linaridins^3, 4^ to highly modified and complex macrocyclic scaffolds. Among the most extensively modified RiPPs are thiopeptides, a class of sulfur-rich, polyazole-containing macrocyclic peptides featuring a central six-membered nitrogen-containing heterocycle^5^. Similar to all RiPPs, thiopeptides are produced from a ribosomally synthesized precursor peptide, involving actions of highly divergent biosynthetic enzymes that are organized in a well-orchestrated process^6–12^. The complex nature of thiopeptide biosynthesis is further demonstrated by the fact that some thiopeptides, such as nocathiacin and nosiheptide, contain additional macrocycle rings that are not derived from the ribosomally synthesized precursor peptides^13–16^.

Nosiheptide (**1**) is a prototypic thiopeptide antibiotic produced by *Streptomyces actuosus* ^17^, which is classified as an e-series thiopeptide, because it contains a hydroxypyridine moiety^5^. This compound blocks bacterial protein translation by targeting the 50S ribosomal subunit^18^ and thereby exhibits highly potent activity against various contemporary pathogens^19^. In addition to the ribosomally derived macrocyclic ring, nosiheptide also possesses a precursor peptide-independent side ring system that mainly consists of an indolyl moiety. This indolyl moiety is produced by a radical *S*-adenosylmethionine (SAM) enzyme NosL, which converts l-Trp to 3-methyl-2-indolic acid (MIA, **3**) via an unusual carbon chain rearrangement. NosL exhibits an astonishing substrate and catalytic promiscuity, and its catalytic mechanism has been investigated in detail^20–30^. It has been shown that the indole side ring is installed before the formation of the thiopeptide-characteristic macrocyclic scaffold^31^, but how MIA is processed and incorporated into nosiheptide biosynthesis remains unknown.

We recently showed that the radical SAM methyltransferase NosN is responsible for attaching a methyl group on the C4 of MIA moiety and this methylation reaction involves a key methylene radical derived from 5′-methylthioadenosine, a SAM cleavage product^32, 33^. The *nosN*-knockout mutant of *S. actuosus* produced a nosiheptide analogue (**2**), which contains an indolyl-*S*-cysteine moiety, whereas the side ring is not formed^14, 32^. This result suggests that the thioester bond formation with the Cys8 thiol likely precedes the ester bond formation with the Glu6 γ-carboxylate. Notably, lactocillin, a thiopeptide produced by a vaginal isolate *Lactobacillus gasseri* JV-V03, contains a different indolyl-*S*-cysteine moiety and does not have a side ring system^34^, demonstrating the diverse biosynthetic chemistry in thiopeptide maturation. In this study, we report investigation of nosiheptide biosynthesis by a series of genetic and biochemical analysis. This investigation revealed the functions and substrate specificities of four enzymes (NosI, NosJ, NosN and NosK) that are involved in the construction of the nosiheptide indole side ring, showing a well-organized biosynthetic process that centers on a previously unknown carrier protein NosJ.

## Results

### NosI is an adenylating enzyme for MIA activation

**Fig. 1:**
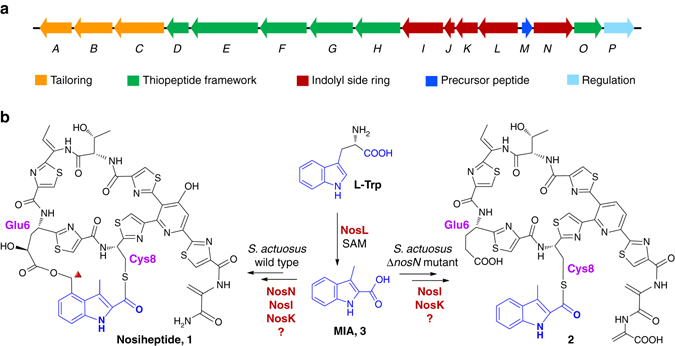
Biosynthesis of nosiheptide by *S. actuosus*. **a** Organization of the nosiheptide biosynthetic gene cluster. Genes involved in the biosynthesis of the indolyl side ring (*nosI*, *nosJ*, *nosK*, *nosL* and *nosN*) are shown in red. **b** Nosiheptide contains an indolyl side ring that is not derived from the precursor peptide NosM. The NosL-derived indolic acid moiety (highlighted in blue) is a major component of the side ring, and the carbon atom introduced by the radical SAM methyltransferase NosN is highlighted with a red triangle. The *nosN*-knockout mutant of *S. actuosus* does not produce nosiheptide but an off-pathway product **2**

**Fig. 2:**
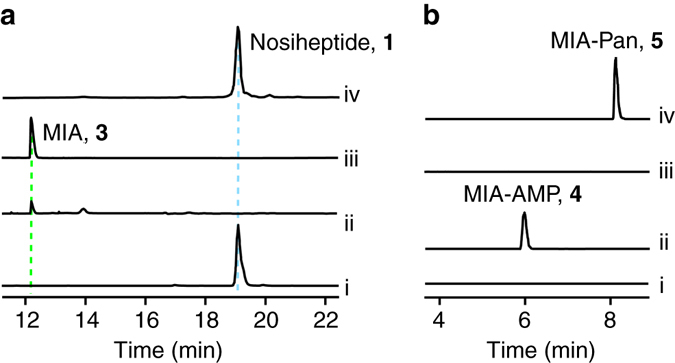
NosI is an adenylating enzyme for MIA activation. **a** HPLC traces of the culture extracts from (i) *S. actuosus* wild-type strain, (ii) the *nosI*-knockout mutant and (iv) the *nosI*-knockout mutant containing a *nosI*-expressing plasmid; MIA synthetic standard (iii) was also analyzed to confirm the production of MIA by the *nosI*-knockout mutant. **b** LC-MS analysis of NosI in vitro activity, showing the extracted ion chromatograms (EICs) of [M+H]^+^  = 505.1 (corresponding to MIA-AMP, **4**) for (i) control reaction with the supernatant of boiled NosI, (ii) NosI reaction with MIA and ATP; and the EICs of [M+H]^+^  = 436.2 (corresponding to the MIA-Pan, **5**) for (iii) control reaction with the supernatant of boiled NosI and (iv) NosI reaction with MIA, ATP, and pantetheine

The nosiheptide gene cluster encodes a putative acyl-CoA ligase NosI (Fig. 1a), which is a possible candidate for MIA activation. However, as proposed previously, it could also be possible that NosI activates the γ-carboxylate of Glu6 and forms the intramolecular ester linkage (Fig. 1b)^14^. Because the *nosN*-knockout mutant strain produces **2**, a nosiheptide analogue whose side ring is not formed (Fig. 1b), we reasoned that if NosI activates the Glu6 γ-carboxylate, a similar side ring-open analogue could be produced by the *nosI*-knockout mutant strain. To test this hypothesis, we knocked out *nosI* from *S. actuosus* by targeted in-frame deletion of the 600-bp internal fragment. High-resolution (HR)-liquid chromatography (LC)-mass spectrometry (MS) analysis showed that nosiheptide production was completely abolished in the mutant (Fig. 2a, trace ii). However, no signal corresponding to a nosiheptide analog could be found. Instead, we observed a compound with a retention time much earlier than that of nosiheptide and a protonated molecular ion at *m*/*z* = 176.0708 (1.7 p.p.m. error for a calculated molecule formula of C_10_H_10_NO_2_) (Fig. 2a, trace ii). This compound was further validated to be MIA by coelution with the synthetic standard (Fig. 2a, trace iii). Introduction of a *nosI*-expressing plasmid into the mutant abolished MIA production and restored nosiheptide production to the wild-type level (Fig. 2a, trace iv). This analysis indicates that NosI may not activate the Glu6 γ-carboxylate; instead, it is likely responsible for MIA activation and incorporation into nosiheptide biosynthesis.

We next expressed NosI in *Escherichia coli* with an N-terminal hexa-histidine tag and purified the protein to near homogeneity by Ni^2+^-affinity chromatography. LC-HR-MS analysis of the reaction mixture containing NosI, MIA and ATP clearly showed the production of a compound with a protonated molecular ion at *m*/*z* = 505.1225, which is absent in the negative control assays (Fig. 2b). The suggested molecule formula C_20_H_21_N_6_O_8_P ([M+H]^+^ calcd 505.1231, 1.2 p.p.m. error) is consistent with **4**, an AMP ester of MIA (Fig. 2b, trace ii), and this is supported by HR-MS/MS analysis (Supplementary Fig. 1). This analysis clearly demonstrated that NosI is an adenylating enzyme responsible for activation of MIA in nosiheptide biosynthesis.

Thioester linkage to the cysteamine group of a phosphopantetheinyl (Ppant) cofactor is a common strategy in biochemistry for activation of a carboxylate. Indeed, addition of pantetheine to the reaction led to production of a compound with a protonated molecular ion at *m*/*z* = 436.1895, which is consistent with **5**, an MIA-pantetheine thioester (MIA-Pan, molecule formula C_21_H_29_N_3_O_5_S, [M+H]^+^ calcd 436.1901, 1.4 p.p.m. error) (Fig. 2b, trace iv) and the identity of **5** was further corroborated by HR-MS/MS analysis (Supplementary Fig. 2). Intriguingly, addition of CoA in the reaction did not produce any detectable amount of MIA-CoA thioester, suggesting that NosI is not a CoA ligase. We also ran the reaction with NosI, MIA, ATP and the precursor peptide NosM. No modification of NosM was observed in this analysis, excluding the possibility that NosI is able to transfer MIA moiety to the precursor peptide NosM. Based on these observations, we proposed that NosI is responsible for MIA adenylation and transferring to an unknown phosphopantetheinylated carrier protein for downstream modifications.

### NosJ is an indolyl carrier protein

**Fig. 3:**
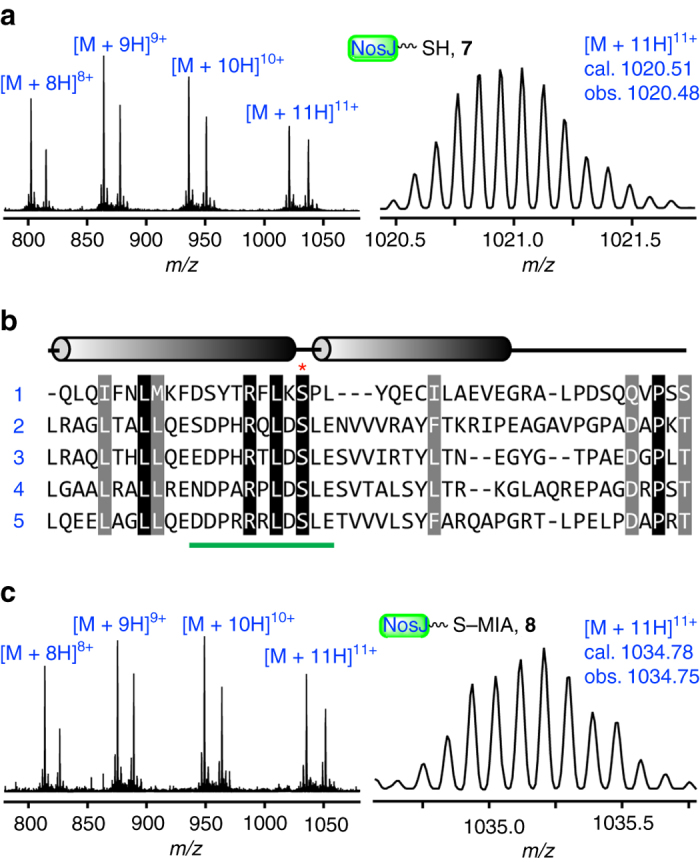
NosJ is a previously unknown carrier protein. **a** HR-MS data of holo-NosJ (**7**) produced from *E. coli* BAP1, a strain that constitutively expresses *sfp*. **b** Sequence alignment of NosJ-homologous proteins, including (**1**) human RGS12 (which is a regulator of G-protein signaling, PDB: 2EBZ_A), (**2**) EPJ36919.1, (**3**) SCF35178.1, (**4**) NocK ADR01086.1 and (**5**) NosJ ACR48339.1. The conserved Ser residues (Ser36 in NosJ) onto which the Ppant arm is attached were shown with a red asterisk. The NosJ_28–38_ Glu_C fragment was shown by a green bar. A NosJ homology model was shown in Supplementary Fig. 4. **c** HR-MS data of NosJ-bound MIA (**8**) produced by incubation of NosJ (100 μM) with 10 μM NosI, 0.5 mM ATP and 0.2 mM MIA for 2 h

Careful examination of the nosiheptide gene cluster led to finding of a small protein NosJ (Fig. 1a), which was previously annotated as a hypothetic protein^14^. To test whether NosJ is a cryptic carrier protein, we expressed NosJ in *E. coli* BAP1^35^, a strain that constitutively expresses a broad-specificity Ppant transferase (PPTase) Sfp from *Bacillus subtilis* ^36^. LC-HR-MS analysis showed that the resulting protein has a molecular weight of 11214.3 Da. (Fig. 3a), which corresponds well to the NosJ holo-form (**7**) that contains a Ppant arm (calcd 11214.5 Da). Sequence alignment of NosJ with several homologous enzymes showed that these enzymes contain a conversed Ser residue (Ser36 in NosJ), which is likely the site at which the PPTase-catalyzed modification occurs (Fig. 3b and Supplementary Fig. 4). To test this hypothesis, NosJ (**7**) was treated with the endoprotease Glu_C and the reaction solution was analyzed by LC-HR-MS. This analysis showed that the proteolytic fragment NosJ_28–38_ indeed contains a Ppant moiety (Fig. 3b and Supplementary Fig. 3), suggesting that the Ppant group is attached to the Ser36 of NosJ. To test whether NosJ is the acceptor of MIA in NosI-catalyzed reaction, we incubated NosJ with NosI, ATP and MIA. LC-HR-MS analysis of the resulting reaction mixture showed that NosJ had a mass increment of 157.0, corresponding well to the NosJ-bound MIA (**8**) (Fig. 3c). Together, these analyses clearly demonstrated that NosJ is a cryptic carrier protein that works together with NosI for the incorporation of MIA into nosiheptide biosynthesis.

### NosJ-bound MIA is the natural substrate of NosN

**Fig. 4:**
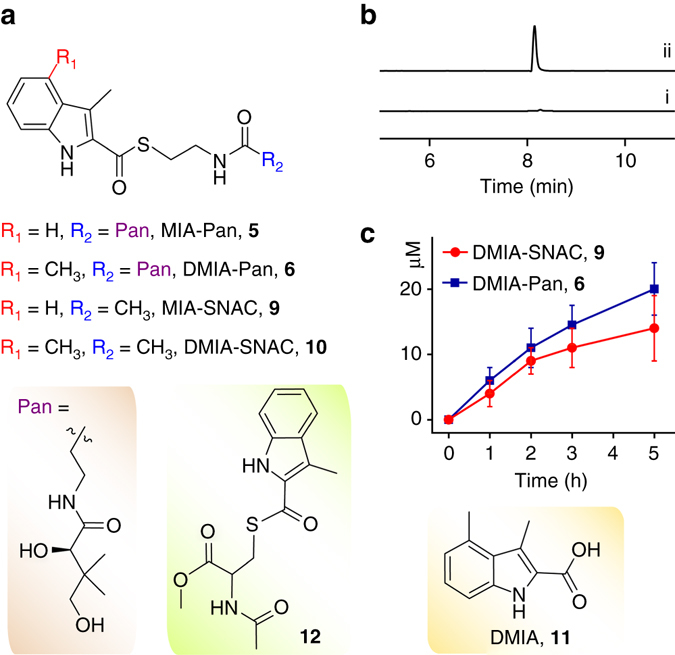
Probing NosN reaction by using substrate analogues. **a** NosN methylates two MIA-based thioester (**5** and **9**) to produce the corresponding indole C4 methylated products (**6** and **10**). **b** Extracted ion chromatograms (EICs) of [M+H]^+^  = 450.2 (corresponding to DMIA-Pan, **6**) for (i) control reaction with the supernatant of boiled NosN, and (ii) overnight reaction with NosN and **5**. **c** Time-course analysis of the NosN reactions with MIA-SNAC **9** and MIA-Pan **5**, showing that **5** is a better substrate of NosN than **9**. The reactions were carried out with 100 μM substrates (**5** or **9**), 1 mM SAM, 1 mM NADPH, 50 µM FldA, 20 µM Fpr and 100 μM NosN. The chemical structures of DMIA (**11**) and **12** are shown; the latter is a structural mimic of the MIA-based thioester associated with a polypeptide Cys residue, which is not a NosN substrate

The methyltransferase activity of the class C radical SAM enzyme NosN has been recently reconstituted in vitro in our lab, which was shown to install a methyl group on the indole C4 of the MIA moiety^32, 33^. Neither MIA (**3**) nor **2** is the NosN substrate, while this enzyme was shown to methylate **9**, an *N*-acetylcysteamine (SNAC) thioester derivative of MIA, to produce **10** (Fig. 4a)^32^. As SNAC serves as a structural mimic of the NosJ Ppant arm, it is likely that the NosN-catalyzed methylation occurs on the NosJ-bound MIA thioester **8**. To validate this hypothesis, we synthesized MIA-Pan (**5**), a good structural mimic of the NosJ-bound MIA thioester **8**, and ran the NosN reaction with **5**. LC-HR-MS analysis clearly showed the production of a compound with a protonated molecular ion at *m*/*z* = 450.2051, which is absent in the control assays (Fig. 4b). This compound is consistent with **6** (Fig. 4a), a pantetheine-bound thioester of 3,4-dimethyl-2-indolic acid (DMIA, **11**) (DMIA-Pan, molecule formula C_22_H_31_N_3_O_5_S, [M+H]^+^ calcd 450.2063, 2.7 p.p.m. error) and the identity of **6** was further corroborated by HR-MS/MS analysis (Supplementary Fig. 5). We next performed the detailed time-course analysis of NosN reactions with **5** and **9**, respectively. This analysis showed that NosN is more efficient with **9** than with **5** (Fig. 4c), supporting that the NosJ-bound MIA thioester **8** is the natural substrate of NosN.

**Fig. 5:**
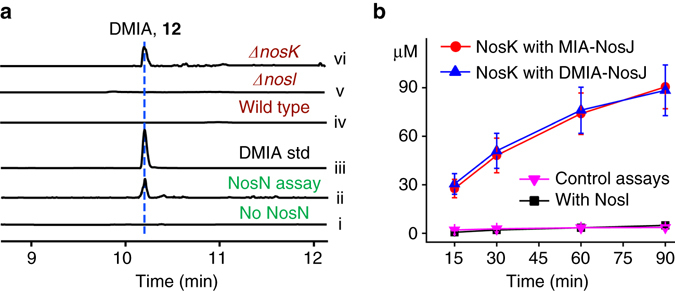
Functional dissection of NosN and NosK. **a** Extracted ion chromatograms (EICs) of [M-H]^−^ = 188.1 (corresponding to DMIA, **11**) for (i) control reaction with the supernatant of boiled NosN, (ii) overnight reaction with 100 μM MIA, 500 μM ATP, 10 μM NosI, 100 μM NosJ and 100 μM NosN that was subsequently treated with 0.2 M NaOH for 30 min, (iii) DMIA synthetic standard and the culture extracts from (iv) *S. actuosus* wild-type strain, (v) the *nosI*-knockout mutant and (vi) the *nosK*-knockout mutant. **b** A time course analysis of NosK-catalyzed MIA and DMIA release from the NosJ-bound thioesters. The reaction was performed by addition of 10 μM NosK or NosI to the solutions containing 100 μM MIA-NosJ or DMIA-NosJ (which were produced by treating apo-NosJ with Sfp and the corresponding CoA thioesters) and the reaction was quenched by addition of an equal volume of methanol at different time points. MIA and DMIA was quantified by HPLC with UV detection at 300 nm. The reaction was performed in triplicates and the SD are shown by the error bars. It should be noted that NosK also hydrolyzes the CoA-bound MIA and DMIA thioesters with comparative efficiencies with that of the NosJ-bound thioesters

To further validate that NosJ-bound MIA **8** is the NosN substrate, we performed a tandem reaction by incubation of NosI, NosJ, NosN and other required components overnight, and the workup was subsequently treated with NaOH to hydrolyze any thioesters. LC-HR-MS analysis of the resulting mixture showed the production of a compound exhibiting a deprotonated molecular ion of 188.0710, which is absent in the negative control reactions (Fig. 5a). The suggested formula C_11_H_11_NO_2_ is consistent with DMIA (**11**) ([M-H]^−^ calcd 188.0711, 0.5 p.p.m. error) and this is further supported by co-elution with the synthetic standard (Fig. 5a, trace iii). These analyses demonstrate that the NosN-catalyzed methylation occurs on the NosJ-bound MIA. It should be noted that NosN does not methylate **12** (Fig. 4), a structural mimic of the Cys-tethered MIA, excluding the possibility that NosN acts on an MIA moiety that is bound to a Cys (or Ser) residue of a polypeptide chain.

### NosK participates in DMIA transfer

*nosK* is co-transcribed with *nosJ* and *nosI*, which encodes a putative hydrolase-like enzyme. Bioinformatical analysis based on sequence similarity network (SSN)^37^ and phylogenetic analysis shows that NosK is evolutionarily related to several hydrolases, including TsrB (39% identity) and TsrU (37% identity) involved in thiostrepton biosynthesis (Supplementary Fig. 6)^13, 38^. To dissect the function of NosK, we knocked out its encoding gene from *S. actuosus* by targeted in-frame deletion of the 405 bp internal fragment. LC-HR-MS analysis showed that nosiheptide production was completely abolished in the *nosK*-knockout mutant and introduction of a *nosK*-expressing plasmid into the mutant restored nosiheptide production to the wild-type level (Supplementary Fig. 7), demonstrating the essential role of NosK in nosiheptide biosynthesis. Intriguingly, careful examination of LC-HR-MS data showed that a compound corresponding well to DMIA **11** was produced by the *nosK*-knockout mutant, which is absent in the cultures of the wild-type and other mutant strains of *S. actuosus* (Fig. 5a), suggesting that NosK is likely involved in transferring DMIA moiety from NosJ to the Cys8 thiol.

To further test this hypothesis, NosK was expressed in *E. coli*, purified to homogeneity and incubated with NosJ-bound DMIA thioester (**13**). A time-course analysis of the resulting reaction clearly showed that DMIA **11** was released from NosJ as a free carboxylate, whereas DMIA production in the parallel control assay with NosI is insignificant (Fig. 5b). These results indicate that DMIA release from NosJ is a NosK-specific reaction, suggesting that NosK participates in transferring the DMIA moiety in nosiheptide biosynthesis. Notably, we found that NosK also releases MIA from NosJ-bound MIA thioester **8** with an efficiency similar to that with **13** (Fig. 5b), suggesting that NosK does not strictly differentiate the indolyl moieties bound to NosJ. We did not observe apparent DMIA transfer from **13** to the precursor peptide NosM (Supplementary Fig. 8) and future studies are awaited to reveal the detailed function of NosK in nosiheptide biosynthesis.

### Kinetic and equilibrium analysis of the NosJ-related interactions

**Fig. 6:**
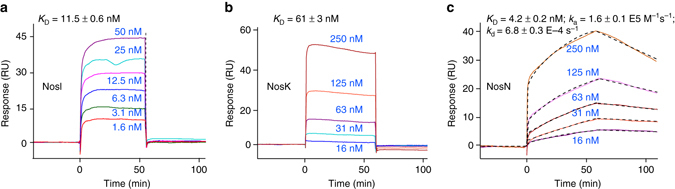
SPR measurements of NosJ binding to its reaction partners. NosI **a** and NosK **b** showed rapid binding and dissociation kinetics, whereas NosN **c** showed a relatively slow kinetics. *K*_D_ represents equilibrium dissociation constant, whereas *k*_a_ and *k*_d_ represent the rate constants of association and dissociation, respectively. Sensorgrams are shown in solid lines with different colors corresponding to different concentrations, and the fittings in **c** are shown in dashed black lines

The results presented above demonstrate that NosJ has a central role in the formation of the nosiheptide indole side ring system. To investigate the possible interactions of NosJ with other proteins, we performed surface plasmon resonance (SPR) analysis using holo-NosJ as a target protein. This analysis showed that NosI, NosN and NosK all bind to holo-NosJ with equilibrium dissociation constants (*K*_D_) in nanomolar range (Fig. 6). NosI and NosK share a similar kinetic behavior of rapid binding and dissociation, and the binding affinity of NosI is ~11-fold higher than NosK (Figs. 6a, b). Intriguingly, the kinetic behavior of NosN is apparently different from those of NosI and NosK (Fig. 6c). NosN exhibits a much slower binding and dissociation process, and have the highest binding affinity with NosJ (*K*_D_ = 4.2 ± 0.2 nM) (Fig. 6c), suggesting that the interaction of NosJ with NosN is more stable than with other enzymes. This result is consistent with the low catalytic efficiency of NosN (Fig. 4c), which appears to be common among the radical SAM superfamily enzymes^39, 40^.

## Discussion

**Fig. 7:**
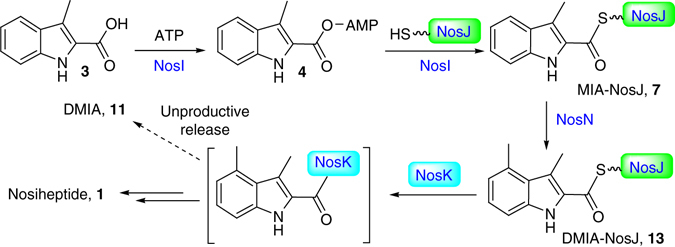
The proposed biosynthetic pathway for MIA incorporation into nosiheptide biosynthesis. The NosN-catalyzed reaction occurs on the NosJ-bound MIA thioester (**7**) and the resulting DMIA moiety is further incorporated into nosiheptide biosynthesis by the α/β hydrolase fold enzyme NosK

Bacterial resistance to currently used antibiotics becomes a growing threat to healthcare and new antibiotics are now in urgent need. Because of the multiple modes of action and the potent activity toward many drug-resistant pathogenic bacteria^5, 18, 41, 42^, thiopeptides serve as a promising group of leads in antibiotic development. Despite significant progress has been made over the past few years^6–12^, many aspects in thiopeptide biosynthesis remain unclear. In this work, we have revealed the function and substrate specificity of NosI, NosJ, NosN and NosK, demonstrating a unique biosynthetic strategy that centers on the Ppant arm of a carrier protein NosJ (Fig. 7). As a similar set of genes are also found in nocathiacin gene cluster (*nocI*, *nocN* and *nocK*, the latter encodes a protein containing both NosK and NosJ homologous sequences)^15^, similar carrier protein-centered pathway is likely involved in the biosynthesis of other e-series thiopeptides.

We first showed that NosI is an adenylating enzyme that activates MIA as an AMP ester (Fig. 7). Intriguingly, panthetheine is an MIA acceptor in NosI-catalyzed reaction, whereas CoA is not, indicating that NosI is not a CoA ligase but likely activates MIA and transfers it to the Ppant arm of a carrier protein. We subsequently validate this hypothesis by showing that NosJ, a previously function unknown protein, is the MIA acceptor in NosI-catalyzed reaction. SPR analysis further validates this proposal, showing a high binding affinity of NosI to NosJ with rapid binding and dissociation kinetics. Further analysis indicated that the Ppant arm is posttranslationally attached to the Ser36 of NosJ, which resides on the junction of two α-helixes as suggested by homology modelling analysis (Fig. 3b and Supplementary Fig. 4).

A unique reaction in nosiheptide side ring formation is the indole C4 methylation catalyzed by the class C radical SAM enzyme NosN^43, 44^. We previously showed that the *nosN*-knockout mutant of *S. actuosus* produced a nosiheptide analog **2** (Fig. 1b), which, unexpectedly, is not the NosN substrate^32^. Instead, NosN methylates the MIA-SNAC thioester **9** to produce DMIA-SNAC **10** ^32^. As SNAC is a Ppant structural mimic, the natural substrate of NosN is likely a Ppant-bound MIA thioester. In this work, we validated this proposal by showing that Pan-MIA (**5**) is even a better substrate of NosN than MIA-SNAC (**9**). As NosN does not methylate **12**, a structural mimic of MIA-Cys thioester, it appears unlikely that NosN-catalyzed reaction occurs after MIA transfer to a Cys (or Ser) residue of a polypeptide (e.g., NosK or the unmodified precursor peptide NosM).

As NosK releases both MIA and DMIA from the corresponding NosJ-bound thioesters with similar efficiencies, it is thus essential to ensure that the NosN-catalyzed methylation proceeds completely before the NosK-catalyzed transferring reaction, because otherwise, the off-pathway product **2** will likely be produced, as in the case of the *nosN*-knockout mutant strain of *S. actuosus*. To this end, NosJ has been endowed with different binding affinities to their reaction partners. The binding affinity of NosJ to NosN is about 30-fold higher than that to NosK, suggesting that NosJ predominantly binds NosN when both NosN and NosK are present. More importantly, unlike NosK that rapidly dissociates from NosJ, dissociation of NosN from NosJ is a relatively slow process (Fig. 6c), suggesting that the NosN–NosJ complex is more stable, which further ensures that the NosN-catalyzed reaction can proceed efficiently. As we did not observe apparent DMIA transfer from NosJ to the precursor peptide NosM in the NosK assay, the acceptor for the DMIA moiety is currently unclear. It also remains elusive how the ester linkage between the Glu6 γ-carboxylate and the DMIA moiety is formed. With the characterization of NosI, NosJ, NosN and NosK in this study, the functions of all the genes in the nosiheptide gene cluster seem clear and gene(s) responsible for forming the Glu6-based ester linkage may possibly reside outside of the nosiheptide gene cluster; this hypothesis awaits future testing.

Another intriguing observation is that a similar indolyl-*S*-cysteine moiety is found in lactocillin, whose biosynthesis involves a similar set of proteins including an adenylating enzyme LclJ, a carrier protein LclI and an alpha-beta hydrolase LclK^34^. However, these enzymes share no sequence similarity with NosI, NosJ and NosK, suggesting that convergent evolution likely has a role in thiopeptide biosynthesis; similar findings have also been made for other RiPPs such as lanthipeptides^45^. The carrier protein-centered pathway revealed by this work may thus pave the way for further biosynthetic investigation of thiopeptides and other RiPP natural products and facilitate bioengineering efforts to generate new peptide natural products.

While this paper was under review, Professor Boal and Professor Booker also reported the successful reconstitution of the NosI- and NosK-catalyzed reactions^46^.

## Methods

### NosN in vitro assays

NosN assays were performed in an anaerobic glove box (Coy Laboratory Product, Inc., USA) with <5 p.p.m. of O_2_. A typical assay was carried out by incubating 100 μM thioether substrate (**5**, **9** or **12**) with 100 μM reconstituted NosN, 1 mM SAM, 1 mM NADPH, 50 µM FldA, 20 µM Fpr in 40 mM Tris-HCl buffer (pH 8.0). Reaction volumes were typically 200 μl and were maintained at room temperature (∼25 °C) and were quenched at different time points by addition of formic acid to a final concentration of 5% (v/v). After removal of the protein precipitates by centrifugation, the supernatant was subjected to LC-HR-MS analysis. The methylated products (**6** and **10**) were quantified by the MS intensities in the LC-HR-MS analysis using 5′-chloro-5′-deoxyadenosine (1 μM) as an internal standard. For time-course analysis, the reaction was performed in triplicates and the standard deviations are shown by the error bars.

### NosK in vitro assays

The NosK reaction was performed by incubation of 100 μM MIA-CoA or DMIA-CoA with ca. 150 μM apo-NosJ (expressed in *E. coli* BL21) with 25 μM sfp for 30 min. Complete reaction of the CoA thioesters and the production of MIA-NosJ or DMIA-NosJ were confirmed by LC-HR-MS analysis. The reaction was started by addition of 10 μM NosK or NosI (negative control) to the solution and the reaction was quenched by addition of an equal volume of methanol at different time points. After removal of the protein precipitates by centrifugation, MIA and DMIA was quantified by high-performance liquid chromatography with UV detection at 300 nm. The reaction was performed in triplicates and the SD are shown by the error bars.

### Surface plasmon resonance

The binding between holo-NosJ and other proteins was evaluated by SPR using a Biacore S200 instrument (GE Healthcare). For these experiments, holo-NosJ containing an N-terminal hexa-His tag was covalently immobilized onto the CM5 sensor chip (GE Healthcare) via amine coupling, and the analyses were performed by running different protein solutions over the chip surface. Holo-NosJ was expressed and purified from *E. coli* BAP1 as described in the Supplementary Methods and the tested proteins was estimated to be of >95% purity by SDS-polyacrylamide gel electrophoresis. The running buffer containing 10 mM Tris-HCl (pH 8.0), 100 mM NaCl, 3 mM EDTA and 0.005% (v/v) Tween-20. Proteins were injected at a flow rate of 30 μl min^−1^ with a contact time of 1 min at 25 °C. The BIAevaluation software 2.0 (GE Healthcare) was used to process the SPR sensorgrams and for curve-fitting to obtain the dissociation constants (*K*_D_) using a 1:1 interaction model.

### Data availability

Data supporting the findings of this study are available within the article and its Supplementary Information and from the corresponding author on request.

## Electronic supplementary material

Supplementary Information
