# Supplementary material for: Biosynthesis of the nosiheptide indole side ring centers on a cryptic carrier protein NosJ
Source: Nat Commun. 2017 Sep 5;8:437. doi: 10.1038/s41467-017-00439-1 (PMC5585349; doi:10.1038/s41467-017-00439-1)
Supplement: Supplementary file 1 — Supplementary Information [file 41467_2017_439_MOESM1_ESM.pdf]

Description of Supplementary Files

File Name: Supplementary Information
Description: Supplementary Figures, Supplementary Tables, Supplementary Methods and Supplementary References

Supplementary Methods

Instrumentation

High-performance liquid chromatography (HPLC) was performed using a Thermo Scientific Dionex Ultimate 3000 system with an evaporative light scattering detector (Alltech, 2000ES) equipped with a DIKMA Diamonsil C18 column (3.5 μm , 150 x 2.1 mm). High resolution mass spectra (HRMS) were acquired using a Q-Exactive™ Focus Hybrid Quadrupole-Orbitrap Mass Spectrometer (Thermo Fisher Scientific Inc.) equipped with a Dionex Ultimate 3000 HPLC system (Thermo Fisher), or an LTQ XL Orbitrap Mass Spectrometer equipped with an Accela 600 U-HPLC system (Thermo Fisher Scientific Inc.). NMR spectra were recorded using Varian Inova 500 MHz NMR spectrometer. UV-vis spectroscopy analysis was performed on a 1900 double beam UV-vis spectrometer (Yoke Instrument Co. Ltd., Shanghai, China). PCR was performed on a Bio-Rad T100™ Thermal Cycler using Phanta® Max Super-Fidelity DNA Polymerase (Vazyme Biotech) or KOD DNA polymerase (TOYOBO).

Chemicals and Biochemicals

All chemical reagents and anhydrous solvents were purchased from commercial sources and used without further purification unless otherwise specified. Most chemicals and biochemicals were purchased for Sigma-Aldrich. Enzymes were from Takara Biotechnology (Dalian, China) or from Vazyme Biotech(Nanjing, China). Biochemicals and culture media were purchased from Sinopharm Chemical Reagent Co. Ltd (China). S-adenosyl-L-methionine (SAM) and kanamycin were purchased from Sangon Biotech Co. Ltd (Shanghai, China). $Fe(NH_4)_2(SO_4)_2{\bullet}6H_2O$ and Na_2S were from Adamas Reagent Co. Ltd (Shanghai, China).

DNA Isolation, Manipulation, and Sequencing.

DNA isolation and manipulation in *E. coli* were carried out according to the standard methods R1. PCR amplifications were carried out on a Bio-Rad T100™ Thermal Cycler using Phanta® Max Super-Fidelity DNA Polymerase (Vazyme Biotech Co. Ltd, China). Primer synthesis and DNA sequencing were performed at the Suzhou Genewiz Biotech Co. Ltd.

Construction of plasmids for overexpressing N-terminally hexa-His-tagged NosI, NosJ, NosK, and NosM

PCR products containing *nosJ*, *nosI*, *nosK*, and *nosM* were each amplified from *Streptomyces actuosus* genomic DNA using the corresponding primer pairs (nosJ-For and nosJ-Rev for *nosJ*, nosI-For and nosI-Rev for *nos*I, nosK-For and nosK-Rev for *nosK*, and nosM-For and nosM-Rev for *nosM*) respectively (see Supplementary Table 2 for the sequence). PCR products were each cloned into the NdeI/XhoI sites of pET28a using the homologous recombination method (ClonExpress II One

Step Cloning Kit Vazyme Biotech Co. Ltd, China) to produce the recombinant constructs pNosI-ET28a, pNosJ-ET28a, pNosK-ET28a, and pNosM-ET28a for expressing *nosJ*, *nosI*, *nosK*, and *nosM* respectively.

Production and Aerobic Purification of NosJ, NosI, NosK,

E. coli BL21 (DE3) cells were transformed via electroporation with the expression plasmids, whereas *E. coli* BAP1[1] was used for expression of holo-NosJ. A single colony transformant was used to inoculate a 5 mL culture of LB supplemented with 100 μg/mL kanamycin. The culture was grown at 37 °C for 12 h and was used to inoculate 1 L of LB medium containing 100 μg/mL kanamycin. Cells were grown at 37 °C and 220 rpm to an OD_{600}～0.6-0.8, and then IPTG was added to a final concentration of 0.1 mM. After additional 18 h of incubation at 22 °C, the cells were harvested by centrifugation at 4000 x g for 15 min at 4 °C. The pellet was used directly for protein purification or stored at −80 °C before use.

Aerobic Protein purification

NosI, NosJ and NosK were purified aerobically. The cell pellet collected by centrifugation was re-suspended in 20 ml lysis buffer (50 mM MOPS, 200 mM NaCl, and 10% glycerol, pH 8.0), and was lysed either by using a high pressure homogenizer (FB-110X, Shanghai Litu Mechanical Equipment Engineering Co., Ltd, China) or by sonication on ice. Cell debris was removed via centrifugation at 21000 x g for 30 min at 4 °C. The supernatant was incubated with 4 ml Ni-NTA resin pre-equilibrated with the lysis buffer, and then subjected to affinity purification on a column. The desired elution fractions were combined and concentrated using an Amicon Ultra-15 Centrifugal Filter Unit, and the concentrated protein solution was desalted using a DG-10 column (Bio-Rad) pre-equilibrated with the elution buffer (50 mM MOPS, 25 mM NaCl, and 10% (v/v) glycerol, pH 8.0). The protein fraction was collected and concentrated, analyzed by SDS-PAGE (10% Tris-glycine gel), and was used directly for in vitro assay or stored at −80 °C upon further use. Protein concentration was determined using a Bradford assay kit (Promega) using bovine serum albumin (BSA) as a standard.

Expression and Purification of the Precursor Peptide NosM

Expression and purification of NosM was performed using a protocol similar to that reported previously.[2] The *nosM*-expressing plasmid was introduced into *Escherichia coli* BL21 (DE3) by electroporation, and a single colony transformant was grown in a shaker in 4 ml of LB medium supplemented with 100 mg/mL kanamycin for 12-15 hours at 37°C. This culture was used to inoculate 2 L of LB/Kanamycin (100 mg/mL) medium in 5L flask shaker, and the culture was grown at

37 °C/200rpm until the OD_{600} reached 0.7-0.9. IPTG was added to a final concentration of 0.5 mM and the culture was grown at 37°C for an additional 3 h. Cells were harvested by centrifugation at 4000 x g for 15 min at 4 °C, and the pellet was resuspended in 20 mL of Buffer A (20 mM NaH_2PO_4, 500 mM NaCl, 0.5 mM imidazole, 10% glycerol, pH 7.5 at 25°C) and lysed by sonication. The sample was centrifuged at 21000 x g for 1hour at 25°C. The pellet was resuspended in 20 mL of denaturing Buffer B (6 M guanidine hydrochloride, 20 mM NaH_2PO_4, 500 mM NaCl, 0.5 mM imidazole pH 7.5 at 25 °C). The insoluble portion was removed by centrifugation (21000 x g for 1hour) and the supernatant was clarified through 0.45-mm syringe filters. The 6 x His NosM peptide was purified by immobilized metal affinity chromatography (IMAC) approach at room temperature using a HiTrap chelating HP nickel affinity column (GE Healthcare). The desired NosM peptide was eluted using 3 x CV (column volume) of Elution Buffer (4M guanidine hydrochloride, 20 mM Tris, pH 7.5 at 25°C, 100 mM NaCl, 1M imidazole) before washing by 20 x CV of Washing Buffer (4M guanidine hydrochloride, 20 mM Tris, pH 7.5 at 25°C, 100 mM NaCl, 50 mM imidazole). Desalting was performed by solid phase extraction (SPE) using BioSelect™ reversed-phase C4 column (214SPE3000, Vydac). Collected fractions were lyophilized to afford white fluffy solid, which was stored at -20°C upon further use.

Preparation of the Reconstituted NosN

Production, purification and reconstitution of NosN were performed according to the previously reported protocol.[3] Protein purification was performed in an anaerobic glove box (Coy Laboratory Product Inc., USA) with less than 5 ppm of O_2. The pellet was resuspended in 30 mL of lysis buffer (40mM Tris, 200 mM NaCl, 10mM imidazole, 10% glycerol, pH 8.0) and was lysed by sonication on ice. Cell debris was removed via centrifugation at 21000 x g for 1 hr at 4 °C. The supernatant was passed through a column containing 4 mL of high-affinity Ni-NTA resin (Qiagen Co. Ltd) pre-equilibrated with lysis buffer, and the column was then washed using 50 mL wash buffer (40 mM Tris, 200mM NaCl, 40 mM imidazole, 10% glycerol, pH 8.0). The protein fractions were collected using 10 mL of elution buffer (40 mM Tris, 200 mM NaCl, 500 mM imidazole, 10% glycerol, pH 8.0). The desired fractions were combined and concentrated using an Amicon Ultra-15 Centrifugal Filter Unit and analyzed by SDS-PAGE (12% Tris-glycine gel).

For reconstitution of the NosN [4Fe-4S] cluster, dithiothreitol (DTT) was added to the purified protein solution to a final concentration of 5 mM. $Fe(NH4)_2(SO4)_2$ solution was then added slowly to a final concentration of 800 μM. After 15min incubation at the room temperature, Na_2S solution was added carefully to a final concentration of 800 μM. After further incubation on ice for 7-10 h, the resulting dark solution was subjected to desalt on a PD-10 (GE) column pre-equilibrated with the elution buffer (40mM Tris, 25mM NaCl, 10 mM DTT and 10% (v/v) glycerol, pH 8.0). The eluted protein fraction

was collected and concentrated, and was used directly for in vitro assay or stored at -80°C upon further use.

Construction of the nosI Mutant

To inactivate *nosI*, a 2.2 kb upstream fragment and 1.8 kb downstream fragment were amplified separately from the *S. actuosus* genomic DNA by PCR using the primer pairs nosI-L-For and nosI-L-Rev, and nosI-R-For and nosI-R-Rev, respectively (Supplementary Table 1). The resulting fragments were purified using a PCR purification kit (CWbiotech Co.Ltd, China) and then cloned into the EcoRI /HindIII site of pKC1139 using ClonExpress MultiS One Step Cloning Kit (Vazyme Biotech Co. Ltd, China) to give the in-frame deletion construct pFDU1711. This plasmid was then introduced into *S. actuosus* via *E. coli-Streptomyces* conjugation. The *nosI* in-frame deletion mutant (designated as mFDU1711) was screened and its genotype was confirmed by PCR and DNA sequencing.

Construction of the nosK mutant

To inactivate *nosK*, a 2.2 kb upstream fragment and 2.1 kb downstream fragment were amplified separately from the *S. actuosus* genomic DNA by PCR using the primer pairs nosK-L-For and nosK-L-Rev, and nosK-R-For and nosK-R-Rev, respectively (Supplementary Table 1). The resulting fragments were purified using a PCR purification kit (CWbiotech Co.Ltd, China) and then cloned into the EcoRI /HindIII site of pKC1139 using ClonExpress MultiS One Step Cloning Kit (Vazyme Biotech Co. Ltd, China) to give the in-frame deletion construct pFDU1713. This plasmid was then introduced into *S. actuosus* via *E. coli-Streptomyces* conjugation. The *nosK* in-frame deletion mutant (designated as mFDU1713) was screened and its genotype was confirmed by PCR and DNA sequencing.

In trans Complementation of nosI in S. actuosus

A ~1.3kb PCR product containing *nosI* was amplified by PCR using the primer nosI-C-For and nosI-C-Rev (Supplementary Table 1), and the resulting DNA fragment was cloned into pMD19-T to yield pNosI-MD19. The 1.3 kb HindIII/XbaI fragment was recovered from pNosI-MD19, which was co-ligated with a 0.4 kb EcoRI/HindIII fragment harboring the promoter PermE* into the EcoRI/XbaI site of pSET152 to yield pFDU1712, in which the 1.3kb fragment containing *nosI* is under the control of the PermE* promoter. pFDU1712 was then introduced into mFDU1711 using *E. coli-Streptomyces* conjugation to yield the recombinant strain mFDU1712.

In trans Complementation of nosK in S. actuosus

A ~0.8 kb PCR product containing *nosK* was amplified by PCR using the primer nosK-C-For and

nosK-C-Rev (Supplementary Table 1), and the resulting DNA fragment was cloned into pMD19-T to yield pNosK-MD19. The 0.8 kb HindIII/XbaI fragment was recovered from pNosK-MD19, which was co-ligated with a 0.4 kb EcoRI/HindIII fragment harboring the promoter PermE* into the EcoRI/XbaI site of pSET152 to yield pFDU1714, in which the 0.8 kb fragment containing *nosK* is under the control of the PermE* promoter. pFDU1714 was then introduced into mFDU1713 using *E. coli-Streptomyces* conjugation to yield the recombinant strain mFDU1714.

Nosiheptide Production and Analysis

Fermentation and production of nosiheptide was performed similarly to a previously reported procedure.[6] 100 μl spore suspension the *S. actuosus* wild type or mutant strains was inoculated into a 2L flask containing 500 ml of seed medium (sucrose 2.0%, corn steep liquor 3.0%, peptone 0.5% and $CaCO_3$ 0.5%) and grown for 36 h at 28 °C, 220 *rpm*. The culture was then transferred into 10 x 500 ml fermentation medium (L-glutamate 0.5%, L-arginine 0.1%, L-aspartate 0.1%, $K_2HPO_4{\cdot}7H_2O$ 0.05%, $MgSO_4{\cdot}7H_2O$ 0.1%, Na_2SO_4 0.26%, $ZnSO_4{\cdot}7H_2O$ 0.001%, $FeSO_4{\cdot}7H_2O$ 0.002%, $CaCO_3$ 0.3%, $MnSO_4{\cdot}H_2O$ 0.002%, and glucose 4%, the pH was adjusted to 7.25 before autoclaving) for continuous cultivation at 28 °C, 220 *rpm* for 7 days. The mycelia cake was collected by centrifugation (4000 x g for 25 min), and was soaked with 3 L acetone overnight, whereas the culture was adjusted to a pH of ~4 before extracted by n-butanol. The organic solvent was removed on a rotary evaporator, and the residue was combined and mixed with 100 ml 100 mM sodium citrate (pH 4.0), and the resulting mixture was extracted three times with an equal volume of ethyl acetate. The organic layer was then combined and taken to dryness on a rotary evaporator, and the residue was redissolved in methanol and analyzed by HPLC. The column (Agilent 1100 with a Zorbax SB-C18 column (9.4 mm × 25 cm) was equilibrated with 85% solvent A (H_2O containing 0.1% TFA) and 15% B (CH_3CN), and developed with the following program: 0 to 3 min, constant 85% A/15% B; 3 to 6 min, a linear gradient from 85% A/15% B to 60% A/40% B; 6 to 12 min, constant 60%A/40% B; 12 to 19 min, a linear gradient from 60% A/40% B to 45% A/55% B; 19 to 22 min, a linear gradient from 45% A/55% B to 15% A/85% B; 22 to 28 min, constant 15% A/85% B; and 28 to 32 min, a linear gradient from 15% A/85% B to 85% A/15% B.

Synthesis

Synthesis of MIA (**3**), MIA-SNAC (**9**), and DMIA (**12**) were detailed in a recent Communication.[3]

Preparation of Methyl N-acetyl-S-(3-methyl-indole-2-carbonyl)cysteinate (11)

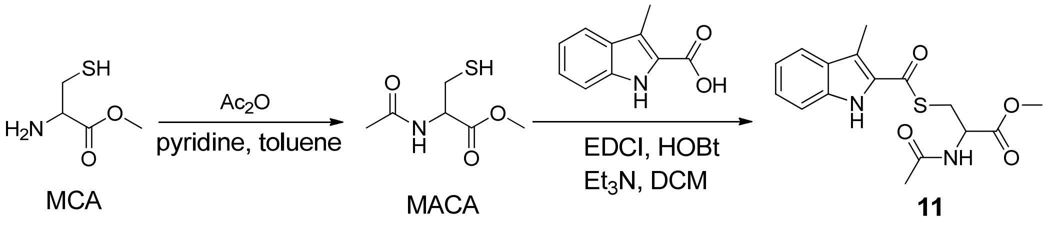

Methyl cysteinate hydrochloride salt (MCA) (1.03 g, 6 mmol) was added to a pyridine-toluene solution (2 mL pyridine in 20 mL toluene), and acetic anhydride (0.56 mL, 7.2 mmol) was subsequently added in a dropwise manner at 0 ^{o}C. After a further stirring for 2 h, the reaction mixture was poured into 30 ml cold water, and toluene was removed by stratification. The aqueous layer was adjusted to pH 7 ~ 8 by saturated $NaHCO_3$, and the resulting solution was then stirred at RT for another 30 min. The mixture was then adjusted to pH 5 ~ 6 by 1M HCl, extracted with AcOEt (20 mL×3), and the combine organic layer was dried by anhydrous $NaSO_4$. The crude methyl N-acetylcysteinate (MACA, 0.85 g) was obtained as yellow oil, which was used for the next step without further purification.

MIA (0.2 g, 1.15 mmol) and MACA (0.35 g, 2 mmol) was dissolved in 20 mL dry DCM. EDCI (0.25 g, 1.3 mmol), HOBt (0.20 g, 1.5 mmol) and Et_3N (0.4 mL) were sequentially added to the solution, and the resulting mixture was stirred at RT overnight. The reaction mixture was poured into 10 ml cold water, extracted with DCM, and the organic layer was washed with water and brine, and dried with anhydrous $MgSO_4$. After removal of the solvent, the crude was purified by silica gel column chromatography to afford pure compound **11** (0.20 g, yield 53%). MS (ESI) $[M+H]^+$: 335.1060 (calculated), 335.1038 (found). ^{1}H NMR (400 MHz, DMSO-d_6) δ 11.63 (s, 1H), 8.56 (d, J = 7.9 Hz, 1H), 7.71 (d, J = 8.1 Hz, 1H), 7.43 (d, J = 8.3 Hz, 1H), 7.31 (t, J = 7.6 Hz, 1H), 7.09 (t, J = 7.5 Hz, 1H), 4.53 (td, J = 8.3, 5.3 Hz, 1H), 3.68 (s, 3H), 3.65 (d, 2H), 2.59 (s, 3H), 1.87 (s, 3H).

Preparation of (R)-pantothenic acid (S2)

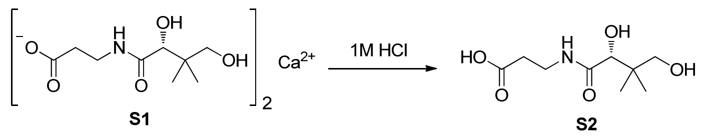

Preparation of (*R*)-pantothenic acid (**S2**) was performed by following a previous report.[7] Briefly, a suspension of calcium (*R*)-pantothenate (**S1**) (25.0 g, 52.6 mmol) in 1M HCl (125 mL) was stirred vigorously until the starting material was completely dissolved. The solution was saturated by the addition of NaCl and the resulting suspension was extracted with EtOAc (6 x 250 mL). The combined

organic layers were dried over Na_2SO_4 and concentrated in vacuo to yield (*R*)-pantothenic acid as a colorless oil (9.5 g, 82%). The crude product was used directly without further purification.

Preparation of (R)-3-(2,2,5,5-tetramethyl-1,3-dioxane-4-carboxamido)propanoic acid (S3)

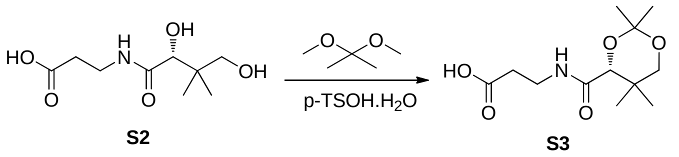

Preparation of **S3** was performed by following a previous report.[8] To a solution of (*R*)-pantothenic acid (**S2**) (9.5 g, 43.4 mmol) in acetone (250 mL), 2-methoxyprop-1-ene (13.5 mL,141 mmol) and pTsOH·H_2O (0.45 g, 2.39 mmol) were added subsequently. The mixture was stirred for 3~4 hours at room temperature and then concentrated in vacuo to give a yellow solid, which was washed with water and acetone. After dried at room temperature, a pale yellow solid was obtained (4.9g, 44%). ^{1}H NMR (DMSO-d6, 400MHz): δ = 0.89 (s, 3H, CH_3), 0.92 (s, 3H, CH_3), 1.38 (s, 6H, -O-C-CH_3), 2.41 (t, 2H, J = 6.8 Hz, -CH_2-COO-), 3.19 (d, 1H, J = 11.6 Hz, -CH_2-O-), 3.21~3.37 (m, 2H, -CH_2-N-), 3.64 (d, 1H, J = 11.6 Hz, -CH_2-O-), 4.04 (s, 1H, -O-CH-CONH-), 7.44 (t, 1H, J = 5.6 Hz, -NH-), 12.26 (s, 1H, -COOH).

Preparation of S4

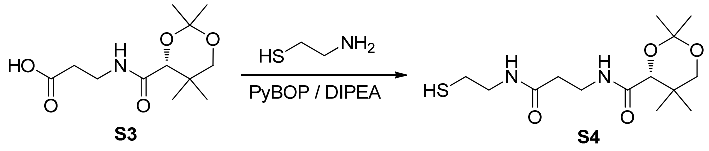

To a solution of acid **S3** (100 mg, 0.39 mmol) in CH_2Cl_2 (2 mL) was added cysteamine hydrochloride (57 mg,0.50 mmol), PyBOP (304 mg, 0.59 mmol) and diisopropylethylamine (0.3 mL, 1.9 mmol). The reaction was allowed to stir for 12 h. The solvent was washed with water (3 x 3 mL). The organic layer were dried over Na_2SO4 and concentrated in vacuo to yield a colorless oil. The crude product was used directly without further purification.

Preparation of S5

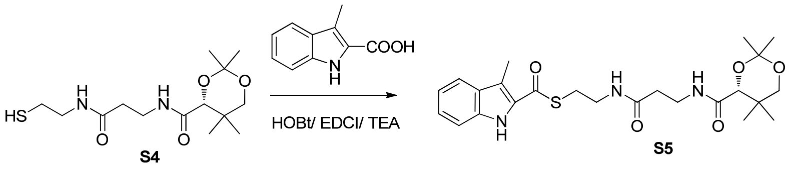

To a solution of crude compound **S4** (0.30 mmol) in CH_2Cl_2 (5 mL) was added MIA (50 mg, 0.282 mmol), HoBt 40.6 mg, 0.30 mmol), EDCI (57.46 mg, 0.30 mmol) and TEA (30.3 mg, 0.30 mmol). The reaction was allowed to stir for 12 h. The solvent was washed with water (3 x 10 mL).

The organic layer were dried over Na_2SO_4 and concentrated in vacuo to yield yellow oil. The crude product was purified using preparative TLC plate (V(Petroleum) / V(ethyl acetate) = 1:3) to give compound **5** (35mg, 24.5%). ^{1}H NMR (DMSO-d6, 400MHz): δ = 0.83 (s, 3H, CH_3), 0.91 (s, 3H, CH_3), 1.36 (s, 6H, -O-C-CH_3), 2.27~2.33 (m, 3H, -$\underline{CH_2}$-CONH-, -S-CH_2-), 2.59 (s, 3H, indol- CH_3), 2.66~2.67 (m, 1H, -S-CH_2-), 2.99~3.03 (m, 1H, -S-CH_2-$\underline{CH_2}$-), 3.14~3.19 (m, 3H, -CH_2-O-, -$\underline{CH_2}$-NHCO-CH-O-), 3.21~3.25 (m, 1H, -S-CH_2-$\underline{CH_2}$-), 3.62 (d, 1H, J = 11.6 Hz, -CH_2-O-), 4.02 (s, 1H, -O-$\underline{CH}$-CONH-), 7.08 (t, 1H, J = 7.6 Hz, indol-H), 7.27~7.31 (m, 1H, indol-H), 7.34~7.41 (m, 1H, indol-H), 7.43 (t, 1H, J = 3.6 Hz, -CONH-), 7.70 (t, 1H, J = 8.8 Hz, indol-H), 8.22 (t, 1H, J = 5.2 Hz, -O-CH-CO$\underline{NH}$-), 11.59 (s, 1H, indol-NH-). HRMS (ESI) m/z $[M+H]^+$: calculated 476.2213, found 476.2219.

Preparation of Compound 5 (in the main text)

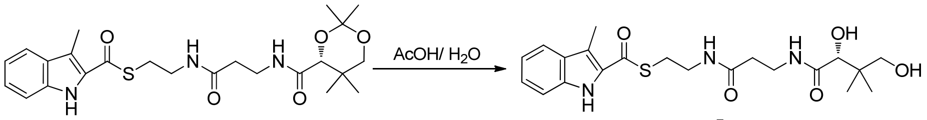

S5 **5**

Compound **S5** was dissolved in AcOH / H_2O (2:1, 2 mL) and the mixture was stirred for 5 h. The solvent was evaporated and the residue was purified using preparative TLC plate (V(Petroleum) / V(ethyl acetate) = 1:3) to give the final product. ^{1}H NMR (DMSO-d6, 400MHz): δ = 0.77 (s, 3H, CH_3), 0.80 (s, 3H, CH_3), 1.22~1.27 (m, 3H, -CH_2-$\underline{OH}$, -NH-CO-$\underline{CH_2}$-), 1.51 (d, 1H, J = 6.8 Hz, -CH-$\underline{OH}$), 2.27~2.33 (m, 3H, -$\underline{CH_2}$-NHCO-CH-OH, -$\underline{CH_2}$-NHCO-), 2.60 (s, 3H, indol-CH_3), 2.67~2.69 (m, 1H, -$\underline{CH_2}$-NHCO-), 3.14~3.18 (m, 4H, -S-CH_2-, -$\underline{CH_2}$-OH), 3.69 (s, 1H, -$\underline{CH}$-OH), 7.07~7.10 (m, 1H, indol-H), 7.28~7.31 (m, 1H, indol-H), 7.43 (d, 1H, J = 8.0 Hz, indol-H), 7.42 (t, 1H, J = 8.0 Hz, -CONH-), 7.69~7.71 (m, 1H, indol-H, -$\underline{NH}$-CO-CH-OH), 8.21 (t, 1H, J = 4.4 Hz, -S-CH_2CH_2-$\underline{NH}$CO-), 11.60 (s, 1H, indol-NH-). HRMS (ESI) m/z $[M+H]^+$: calculated 436.1896, found 436.1906.

Synthesis of CoA thioesters

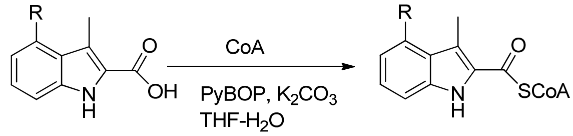

R = H or CH_3 R = H or CH_3

Under the protection of N_2, 50 μmol MIA or DMIA, 30 mg PyBOP and 15 mg K_2CO_3 were

dissolved in 2 ml THF in a 15 mL serum bottle, and 39 μm CoA tri-lithium salt dissolved in 1 ml ddH_2O was added to the solution. The reaction mixture was stirred at room temperature for 3 hrs, and was then filtered using 0.45μm filter membrane. The resulting CoA thioester was purified from the resulting solution using semi-preparative HPLC, which was carried out on a Thermo Scientific Dionex Ultimate 3000 system with UV detection at 254 nm and a flow rate of 2.5 $mL \cdot min^{-1}$. The C18 semi-preparative column (Kromasil C18, 10 x 250 mm, 5 μm) was equilibrated with 85% solvent A (H_2O containing 10 mM NH_4OAc) and 15% B (Methanol), and developed with the following program: 0 to 20 min, a linear gradient from 85% A/15% B to 15% A/85% B; 20 to 22 min, a linear gradient from 15% A/85% B to 85% A/15% B. CoA thioesters were produced as a white powder after lyophilization of the desired elution collected around 11-12 min. ESI-MS ($[M+H]^+$) MIA-CoA: 925.1753 (calc.), 925.1761 (found); DMIA-CoA: 939.1901 (calc.), 939.1889 (found)

Supplementary Figure 1. HR-MS/MS analysis of MIA-AMP (**4**), showing the collision induced dissociation (CID) fragments and the MS/MS spectrum.

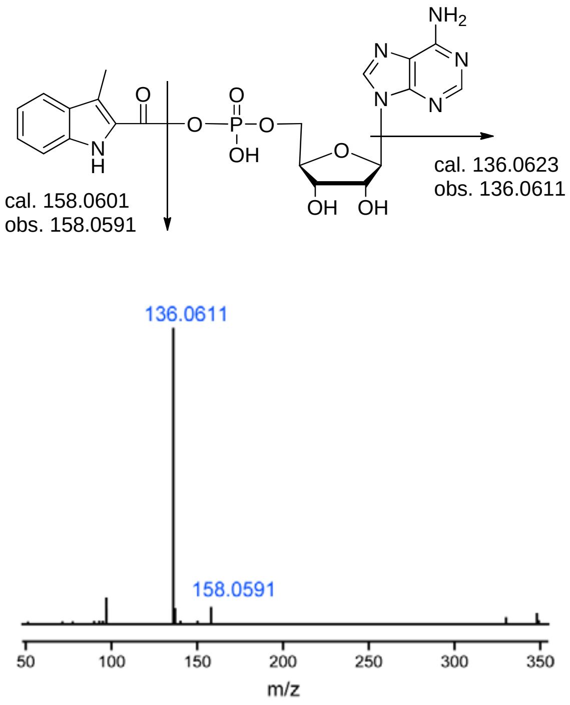

Supplementary Figure 2. HR-MS/MS analysis of MIA-Pan (**5**) produced in the NosI reaction with MIA, ATP and pantetheine, showing the collision induced dissociation (CID) fragments and the MS/MS spectrum.

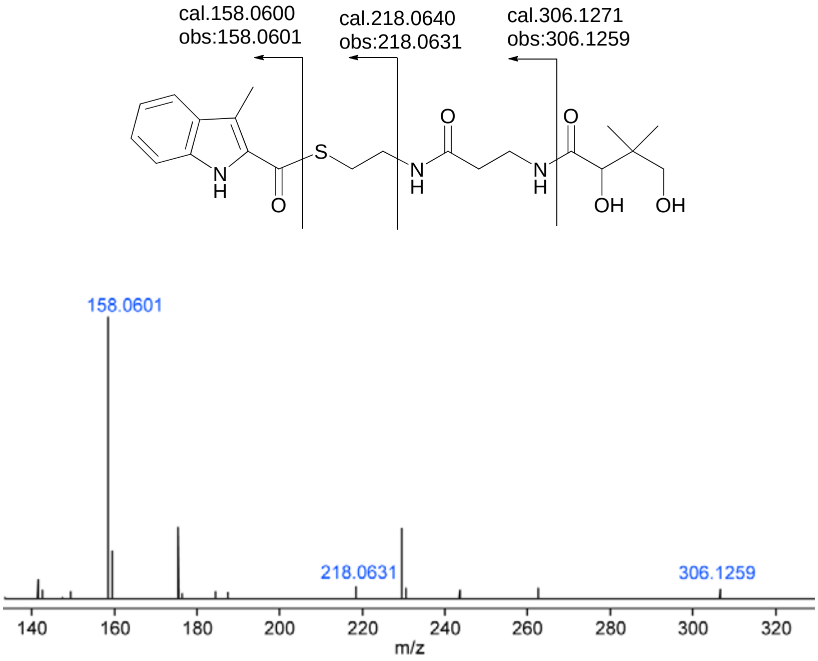

Supplementary Figure 3. HR-MS spectrum of $NosJ_{28-38}$ obtained by GluC-digestion of NosJ, which contains a Ppant arm on Ser36.

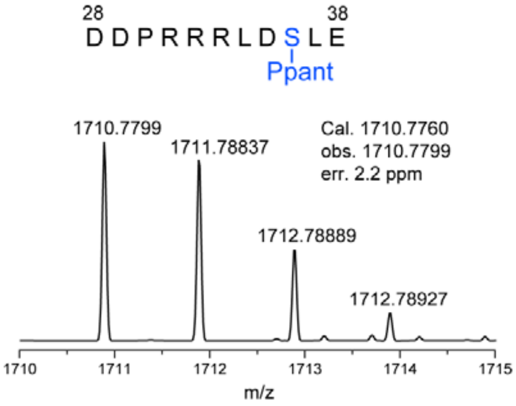

Supplementary Figure 4. Homology modeling of NosJ using SWISS Model[9] based on the structure of human RGS12 (PDB: 2EBZ_A). (A) The sequence alignment of NosJ with RGS12 (24% identity, 31% similarity). The secondary structure predicted by JPred4[10] is shown above the NosJ sequence (blue font, H indicates helix). This analysis is basically consistent with that predicted by SWISS Model, and the only difference lies between Pro30 and Leu34 (highlighted in yellow), which is likely because Pro30 disrupts the α-helical structure. (B) The NosJ homology structure. The Ppant arm is attached to Ser36, the non-hydrogen atoms of which are shown in spheres. The Local quality estimate (C) and the QMean score (D) of the SWISS Model prediction are also shown.

A

```
          HHHHHHHHHHHHHHH-------HHHHHHHHHHHHHH-----------HHHHHHHHHHHHHHHHH---
NosJ      DLQEELAGLLQEDDPRRRLDSLETVVVLSYFARQAPGRTLPELPDAPRTIEGWVTWADQRSSAS
2ebz      -QQLQIFNLMKFDSYTRFLKSPL---YQECILAEVEGRALPDSQQVPSS---------------
```

B

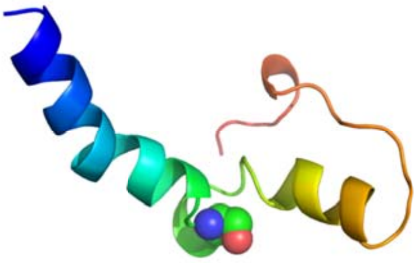

C

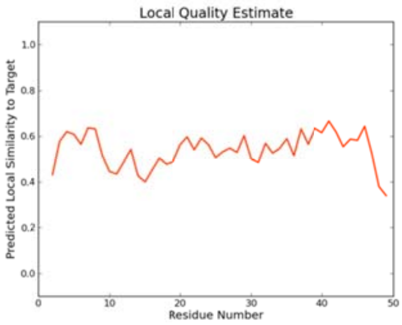

D

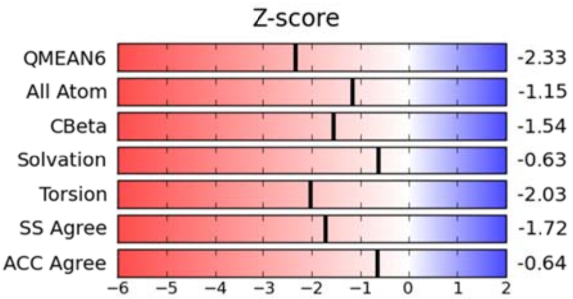

Supplementary Figure 5. HR-MS/MS analysis of DMIA-Pan (**6**), showing the collision induced dissociation (CID) fragments and the MS/MS spectrum.

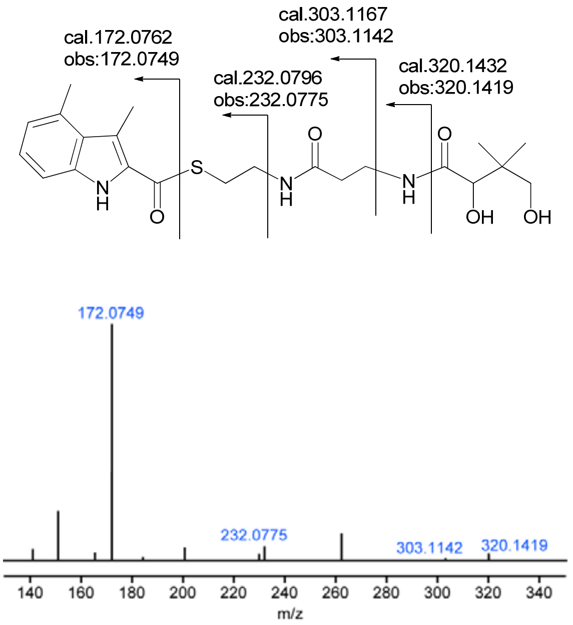

Supplementary Figure 6. Bioinformatic analysis of NosK analogous enzymes, showing (A) the Maximum-likelihood phylogeny, and (B) the sequence similarity network (SSN). The accession numbers of the associated organisms of these NosK analogous enzymes are summarized in Supplementary Table 3.

A

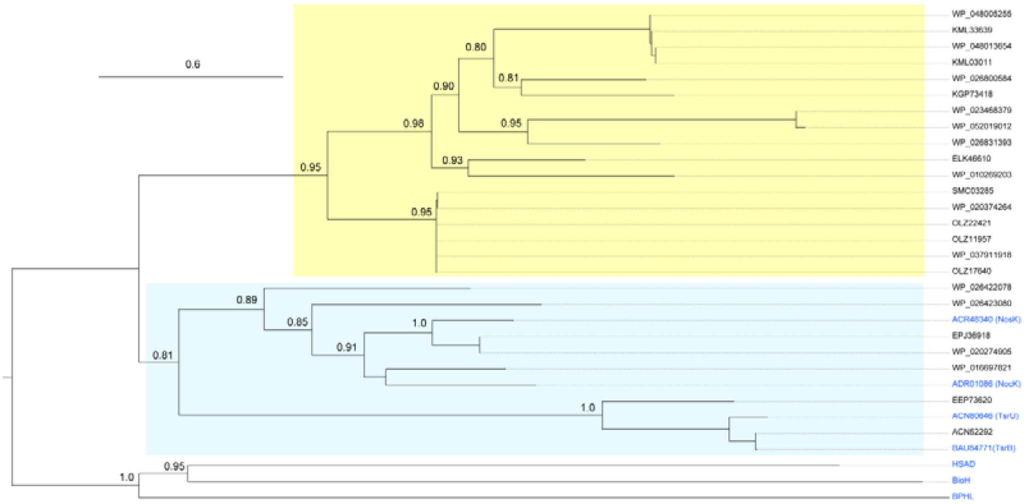

B

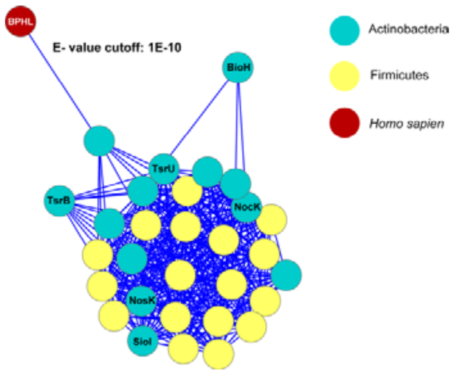

Supplementary Figure 7. NosK is essential for nosiheptide biosynthesis, showing the HPLC traces of the culture extracts from *S. actuosus* wild type strain, the *nosK*-knockout mutant, and the *nosK*-knockout mutant containing a *nosK*-expressing plasmid. DMIA production was not observable in HPLC chromatogram and only observed in LC-MS analysis (see Fig. 5a in the main text).

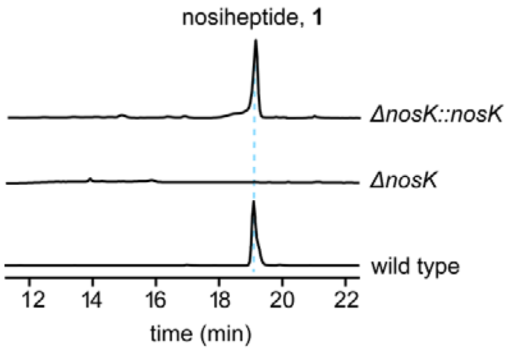

Supplementary Figure 8. MS spectrum of NosM treated with NosK and DMIA-NosJ, showing that NosM was not modified. The sequence of NosM is GSSHHHHHHS SGLVPRGSHM DAAHLSDLDI DALEISEFLD ESRLEDSEVV AKVMSASCTT CECCCSCSS.

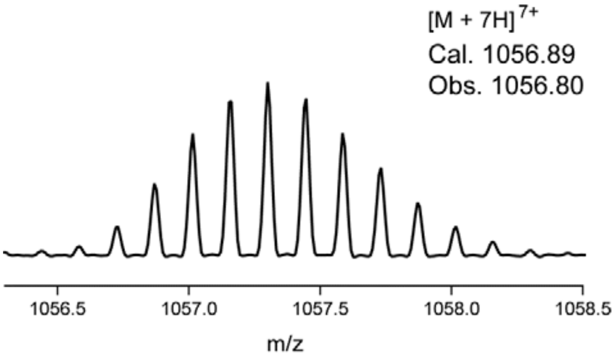

Supplementary Table 1. PCR primers used in this study.

Primer	Sequence	Function
nosJ-For	GTGCCGCGCGGCAGCCATATG ACATCTCAGCGCACCACACCCC	NosJ overexpression
nosJ-Rev	GTGGTGGTGGTGGTGCTCGAGTCAGGAAGCGCTGGAACGCTGG	NosJ overexpression
nosI-For	GTGCCGCGCGGCAGCCATATG GGTGACATGGGCCGACCAGCG	NosI overexpression
nosI-Rev	GTGGTGGTGGTGGTGCTCGAGTCAGAGCTTCCCGTGCGCGCTGT	NosI overexpression
nosK-For	GTGCCGCGCGGCAGCCATATGGACGCGGAGACGCCGATGGACAC	NosK overexpression
nosK-Rev	GTGGTGGTGGTGGTGCTCGAGTCACGTGTCGCCCTTCTGCGTCG	NosK overexpression
nosM-For	GTGCCGCGCGGCAGCCATATG GACGCTGCACACCTGTCCGACC	NosK overexpression
nosM-Rev	GTGGTGGTGGTGGTGCTCGAG TCAGGAGGAGCAGGAACAGCAG	NosK overexpression
nosI-L-For	CATGATTACGAATTCGCACACCCGGCTCGCCTCCGCCTTC	*nosI* in-frame deletion
nosI-L-Rev	GTCGAAGCGGCCGTAGGGGCCGACGGCCACCTTGGGCGC	*nosI* in-frame deletion
nosI-R-For	GTGGCCGTCGGCCCCTACGGCCGCTTCGACGCCGACGGCAA	*nosI* in-frame deletion
nosI-R-Rev	GCCAGTGCCAAGCTTCGAGCGCCGCGTGCAGCCGGGCGCGC	*nosI* in-frame deletion
nosI-C-For	TCCAAGCTT GTGGGTGACATGGGCCGACCAGCG	Δ*nosI* complementation
nosI-C-Rev	TGGTCTAGA TCAGAGCTTCCCGTGCGCGCTGTAG	Δ*nosI* complementation
nosK-L-For	CATGATTACGAATTCGGATGTCACCTGCCGCGCGATGCAT	*nosK* in-frame deletion
nosK-L-Rev	CTTCAGGGAGCCGTTGTGACCGGGCAGATCCACGGGTACG	*nosK* in-frame deletion
nosK-R-For	GATCTGCCCGGTCACAACGGCTCCCTGAAGTCCGTCGAAC	*nosK* in-frame deletion
nosK-R-Rev	GCCAGTGCCAAGCTTTGGCGTACCGCTCGCGCATCCAGCC	*nosK* in-frame deletion
nosK-C-For	TCCAAGCTT GTGGACGCGGAGACGCCGATGGACAC	Δ*nosK* complementation
nosK-C-Rev	TGGTCTAGA TCACGTGTCGCCCTTCTGCGTCGTTC	Δ*nosK* complementation

Supplementary Table 2 Bacteria strains and plasmids used in this study.

Stain/Plasmid	Relevant genotype/comments	Reference
**Strain**		
Escherichia coli DH5α	Host for general cloning	Invitrogen
E. coli BL21 (ED3)	Host for protein expression	Invitrogen
E. coli BL BAP1		(1)
Streptomyces actuosus ATCC 25421	Nosiheptide producing strain	ATCC
mFDU1711	*nosI* in-frame deletion mutant of *S. actuosus*	This study
mFDU1712	*nosI* complemented to mFDU1711	This study
mFDU1713	*nosK* in-frame deletion mutant of *S. actuosus*	This study
mFDU1714	*nosK* complemented to mFDU1713	This study
**Plasmid**		
pET28a	Vector for expression of N-terminal 6 X His-tagged protein in *E.coli*	Novagen
NosN-ET28a	pET28a derivative, containing the a 1.2 kb PCR product encoding NosN	(3)
NosI-ET28a	pET28a derivative, containing the a 300 bp PCR product encoding NosI	This study
NosJ-ET28a	pET28a derivative, containing the a 1.3 kb PCR product encoding NosJ	This study
NosK-ET28a	pET28a derivative, containing the a 0.8 kb PCR product encoding NosJ	This study
pNosI-MD19	pMD19-T derivate, containing 1.3kb PCR product encoding NosI	This study
pNosK-MD19	pMD19-T derivate, containing 0.8 kb PCR product encoding NosK	This study
pFDU1711	*nosI* in-frame deletion construct	This study
pFDU1712	*nosI* complementation construct	This study
pFDU1713	*nosk* in-frame deletion construct	This study
pFDU1714	*nosk* complementation construct	This study

Supplementary Table 3 The accession number and organisms of the NosK homologues enzymes discussed in Supplementary Figure 7.

Organism	Candidate	Annotation
Phylum Actinobacteria<br>Class Streptomycetales<br>*Streptomyces actuosus*	ACR48340	Putative hydrolase
Streptomyces afghaniensis 772	EPJ36918	Putative hydrolase
Streptomyces afghaniensis	WP_020274905	Putative hydrolase
Streptomyces laurentii	ACN52292	TsrU
Streptomyces sioyaensis	ACN80646	SioU
Streptomyces laurentii	BAU84771	TsrB
Streptomyces sp. IB2014 011-1	ONI54807	Pimeloyl-[acyl-carrier protein] methyl ester esterase
Class Pseudonocardiales<br>*Actinoalloteichus spitiensis*	WP_016697821	hypothetical protein
Actinokineospora inagensis	WP_026422078	hypothetical protein
Actinokineospora inagensis	WP_026422080	alpha/beta hydrolase
Class Corynebacteriales<br>*Nocardia* sp. ATCC 202099	ADR01086	NocK
Rhodococcus jostii RHA1	HSAD_RHOJR	Putative hydrolase
Class Micromonosporales<br>*Micromonospora* sp. ATCC 39149	EEP73620	hypothetical protein
Phylum Firmicutes<br>Class Clostridia<br>*Sulfobacillus thermosulfidooxidans*	OLZ11957	hypothetical protein
Sulfobacillus thermosulfidooxidans	OLZ17640	hypothetical protein
Sulfobacillus thermosulfidooxidans	OLZ22421	hypothetical protein
Sulfobacillus thermosulfidooxidans DSM 9293	SMC03285	Pimeloyl-ACP methyl ester carboxylesterase
Sulfobacillus thermosulfidooxidans	WP_037911918	alpha/beta hydrolase
Sulfobacillus thermosulfidooxidans	WP_020374264	alpha/beta hydrolase
Class Bacilli<br>*Bacillus marisflavi*	WP_048013654	hypothetical protein
Bacillus marisflavi	KML03011	hypothetical protein
Bacillus marisflavi	KML33639	hypothetical protein
Bacillus marisflavi	WP_048005255	hypothetical protein
Pontibacillus halophilus	WP_026800584	alpha/beta hydrolase
Paenibacillus senegalensis	WP_010269203	alpha/beta hydrolase
Halobacillus sp. BAB-2008	ELK46610	alpha/beta hydrolase
Exiguobacterium antarcticum	WP_026831393	alpha/beta hydrolase
Exiguobacterium sp. MH3	WP_023468379	hypothetical protein
Exiguobacterium sp. RIT341	WP_052019012	hypothetical protein
Pontibacillus yanchengensis Y32	KGP73418	alpha/beta hydrolase
Phylum Chordata, Class mammalia<br>*Homo sapiens*	BPHL_HUMAN	Valacyclovir hydrolase

Supplementary **References**

1. Pfeifer, B.A., Admiraal, S.J., Gramajo, H., Cane, D.E. & Khosla, C. Biosynthesis of complex polyketides in a metabolically engineered strain of E. coli. *Science* **291**, 1790-1792. (2001).
2. Li, B., Cooper, L.E. & van der Donk, W.A. In vitro studies of lantibiotic biosynthesis. *Methods Enzymol.* **458**, 533-558 (2009).
3. Ding, W. et al. The Catalytic Mechanism of the Class C radical S-Adenosylmethionine Methyltransferase NosN. *Angew Chem Int Ed* **56**, 3857-3861 (2017).
4. Yu, Y. et al. Nosiheptide Biosynthesis Featuring a Unique Indole Side Ring Formation on the Characteristic Thiopeptide Framework. *ACS Chem. Biol.* **4**, 855-864 (2009).
5. Guo, H. et al. Insight into bicyclic thiopeptide biosynthesis benefited from development of a uniform approach for molecular engineering and production improvement. *Chem Sci* **5**, 240-246 (2014).
6. Houck, D.R., Chen, L.C., Keller, P.G., Beale, J.M. & Floss, H.G. Biosynthesis of the modified peptide antibiotic nosiheptide in Streptomyces actuosus. *J. Am. Chem. Soc.* **110**, 5800-5806 (1988).
7. Jansen, P.A. et al. Discovery of small molecule vanin inhibitors: new tools to study metabolism and disease. *ACS Chem Biol* **8**, 530-534 (2013).
8. Agarwal, V. et al. Chemoenzymatic Synthesis of Acyl Coenzyme A Substrates Enables in Situ Labeling of Small Molecules and Proteins. *Org Lett* **17**, 4452-4455 (2015).
9. Biasini, M. et al. SWISS-MODEL: modelling protein tertiary and quaternary structure using evolutionary information. *Nucleic Acids Research* **42**, W252-W258 (2014).
10. Drozdetskiy, A., Cole, C., Procter, J. & Barton, G.J. JPred4: a protein secondary structure prediction server. *Nucleic Acids Res* **43**, W389-394 (2015).
